# Regulation of the Ni^2+^ Content in a Hierarchical Urchin-Like MOF for High-Performance Electrocatalytic Oxygen Evolution

**DOI:** 10.3389/fchem.2019.00411

**Published:** 2019-06-05

**Authors:** Yijian Tang, Shasha Zheng, Huaiguo Xue, Huan Pang

**Affiliations:** School of Chemistry and Chemical Engineering, Guangling College, Yangzhou University, Yangzhou, China

**Keywords:** Ni/Zn-MOFs, oxygen evolution reaction, electrocatalysis, hierarchical urchin-like, hydrothermal method

## Abstract

The exploitation of efficient non-precious electrocatalysts for the oxygen evolution reaction is extremely important but remains tremendously challenging. Here, we prepared a series of hierarchical urchin-like bimetallic Ni/Zn metal-organic framework nanomaterials that served as high-performance electrocatalysts, by regulating the Ni^2+^/Zn^2+^ ratio and using a facile one-step hydrothermal method for the application of the oxygen evolution reaction. The structure of the hierarchical urchin-like microspheres could improve the utilization efficiency of the active species by facilitating the diffusion of gas and reducing the transport resistance of ions, due to its features of a large interfacial area and convenient diffusion channels. In addition, we found that the higher the Ni ratio was, the better the electrocatalytic performance of these bimetallic metal-organic framework nanomaterials.

## Introduction

Efficient and sustainable energy storage and conversion devices, such as water splitting, fuel cells, and metal-air batteries, are currently being extensively researched (Yan et al., 2016; Zhao et al., 2016; Xu H. et al., 2018). The oxygen evolution reaction (OER) is a crucial process for many applications of energy conversion (Nai et al., 2017; Yan D. et al., 2017; Zhao et al., 2017; Zhu et al., 2018; Li X. et al., 2019). To date, RuO_2_ and IrO_2_ are two standard OER catalysts because of their high catalytic activity (Wu et al., 2017; Zhou et al., 2019). However, the low abundance of Ru and Ir makes it impossible to utilize them on a massive scale (Li et al., 2017; Wang M. et al., 2018; Wang X. et al., 2018). Therefore, extensive attention has been paid to exploring non-noble catalysts with excellent stability and activity (Feng et al., 2016a,b; Yan L. et al., 2017; Wang X. et al., 2018; Xu Y. et al., 2018; Huang et al., 2019;Wang et al., 2019).

Metal-organic frameworks (MOFs), which are constructed from the coordination of metal ions with organic ligands, are considered as a type of porous versatile material that can be used for a wide range of applications (Yu F. et al., 2016; Liu et al., 2017; Shi et al., 2017; Zheng et al., 2017), including their promising application to the OER (Zheng S. et al., 2018). In practice, the reactive centers of MOFs themselves are regarded as the metal sites at the anodes of MOF. Therefore, transition-metal based MOFs can be readily applied to OER processes (Yu X. Y. et al., 2016; Zhao et al., 2016). Compared with single metal MOF nanomaterials, bimetallic MOF nanomaterials have displayed excellent electrocatalytic activities because of the synergetic effects between distinct metals. Ni/Fe-based nanosheets (Li F. L. et al., 2019), Ni/Co-based hollow arrays (Song et al., 2019), and Ni/Cu-based nanosheets (Zheng X. et al., 2018) have been reported as good catalysts for the OER. Among these electrocatalysts, many bimetallic MOF catalysts have exhibited exceptional catalytic properties, and many bimetallic systems have shown promising prospects for the application (Lu et al., 2017). However, the stability problem of MOFs may hinder their long-term use and widespread applications (Wang X. et al., 2018). Coincidentally, the urchin-like structure of MOFs could promote the stability of electrocatalysts. This shape can be helpful for improving the utilization efficiency of active species by accelerating gas diffusion and shortening ion transport resistance, owing to its large interfacial area and convenient diffusion channels (Xu et al., 2016; You et al., 2016; Deng et al., 2017).

Herein, a series of hierarchical urchin-like Ni/Zn bimetallic MOF nanomaterials, which acted as efficient electrocatalysts for the OER, were prepared by a facile one-step hydrothermal strategy. Through regulation of the Ni/Zn ratio, the structure of the hierarchical urchin-like MOF becomes increasingly uniform as the Ni content increases, resulting in the high electrocatalytic performances of these bimetallic MOF nanomaterials. This work will promote the development of hierarchical urchin-like MOFs as promising electrocatalysts. In addition, the synergistic effects of Zn^2+^ and Ni^2+^, which contributed to the high electrochemical performance, should be further explored.

## Results and Discussion

**Figure 1:**
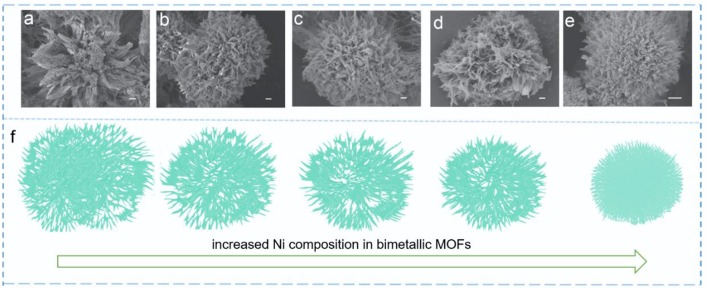
SEM (scale bar 500 nm) images of **(a)** K1, **(b)** K2, **(c)** K3, **(d)** K4, and **(e)** K5. **(f)** Morphological changes of the bimetallic Ni/Zn MOFs with increasing Ni content.

A series of bimetallic Ni/Zn MOF nanomaterials (K1-K5, where the content of Ni increases from K1 to K5) were prepared through a facile hydrothermal method from the coordination of PTA and Ni^2+^/Zn^2+^. As shown in Table S1, the molar ratios of the bimetallic ions (Ni^2+^/Zn^2+^) in the MOFs and reactants are demonstrated. This clearly reveals that the Ni^2+^/Zn^2+^ ratio in these MOFs can be easily regulated by adjusting the Ni^2+^/Zn^2+^ ratio in the reactants. Scanning electron microscopy (SEM) and transmission electron microscopy (TEM) were used to show the morphologies and microstructural features of these samples. The low-magnification SEM images present the fairly dispersed urchin-like microspheres (Figure S1). The high-magnification SEM images clearly show the morphologies of the hierarchical urchin-like shapes (Figure 1). As shown in Figure 1e, the K5 sample presents an urchin-like hierarchical microsphere with a size of 4–5 μm, which is smaller than that of the other samples. This shows that the size of the urchin-like microspheres decreases with increasing Ni content in the bimetallic Ni/Zn MOFs. The urchin-like microspheres are composed of radially oriented nanobelts that become increasingly uniform with increasing Ni content (Figure 1f).

**Figure 2:**
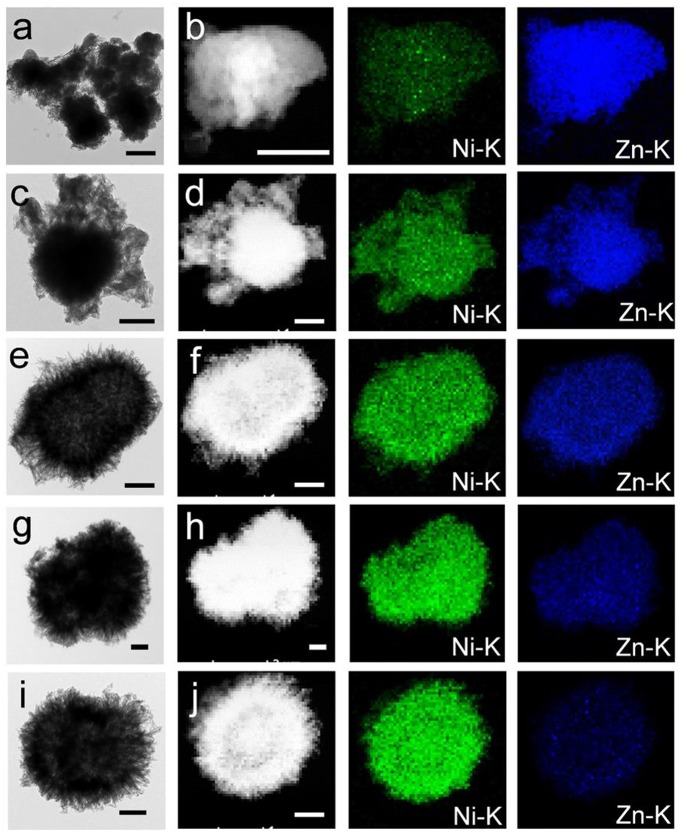
TEM (scale bar 500 nm) images of **(a)** K1, **(c)** K2, **(e)** K3, **(g)** K4, and **(i)** K5. Elemental mapping (scale bar 100 nm) images of **(b)** K1, **(d)** K2, **(f)** K3, **(h)** K4, and **(j)** K5.

Moreover, as the Ni content is increased, the size of the hierarchical urchin-like microspheres become smaller and smaller. The microspheres are more uniform and their nanobelts are longer with increasing Ni content, which are conducive to ion diffusion (Figure S2, Figures 2a,c,e,g,i). The Zn and Ni species are uniformly distributed in each of the K1-K5 samples, which were demonstrated by the elemental mapping (Figures 2b,d,f,h,j). To further accurately verify the Ni/Zn ratio, the elemental components of the K1-K5 samples were assessed by using energy-dispersive X-ray spectroscopy (Figure S3). As depicted in Table S1, the Ni^2+^/Zn^2+^ ratio was further confirmed by inductively coupled plasma optical emission spectrometry. The X-ray diffraction (XRD) patterns obviously reveal the crystal and phase structure of the bimetallic Ni/Zn MOFs. As depicted in Figure S4, the XRD patterns are in agreement with previously reported patterns in the literature, and the MOFs have a formula of [Ni_3_(OH)_2_(C_8_H_4_O_4_)_2_(H_2_O)_4_]·2H_2_O (Yang et al., 2014). The Fourier transform infrared (FTIR) spectra (Figure S5) of these bimetallic MOF nanomaterials is demonstrated. The stretching modes of OH^−^ lead to bands appearing at 3,608 cm^−1^. A strong peak appears at 3,337 cm^−1^, which implies the presence of coordinated H_2_O molecules within these bimetallic MOF nanomaterials. Moreover, the band at 1,507 cm^−1^ results from the stretching modes of the para-aromatic CH groups. In addition, bands appear at 1,572 and 1,382 cm^−1^, which arise from the symmetric and asymmetric stretching modes of the coordinated groups (-COO^−^). Although the FTIR spectra of K1-K5 have practically similar peak positions, the separation of the wave number between the symmetric and asymmetric stretching modes of the -COO^−^ groups increases slightly as the Ni^2+^ ion content increases in the bimetallic MOF nanomaterials, suggesting that the doping of Ni has some influence on the structures of these bimetallic MOF nanomaterials.

X-ray photoelectron spectroscopy was used to analyze the surface electronic states and the chemical compositions of these bimetallic MOF nanomaterials. Two major peaks (Ni 2p_3/2_ and Ni 2p_1/2_), as shown in Figure S6, were detected in the K1-K5 samples. Combined with the results in Figure S7, the binding energy of Ni 2p_3/2_ increases and the binding energy of Zn 2p_3/2_ decreases after hybridization (the binding energies of Ni 2p_3/2_ and Zn 2p_3/2_ in the Ni/Zn MOFs are ~855.9 and 1021.4 eV, respectively). From the above, the Zn^2+^ and Ni^2+^ have a strong interaction in these Ni/Zn MOFs.

**Figure 3:**
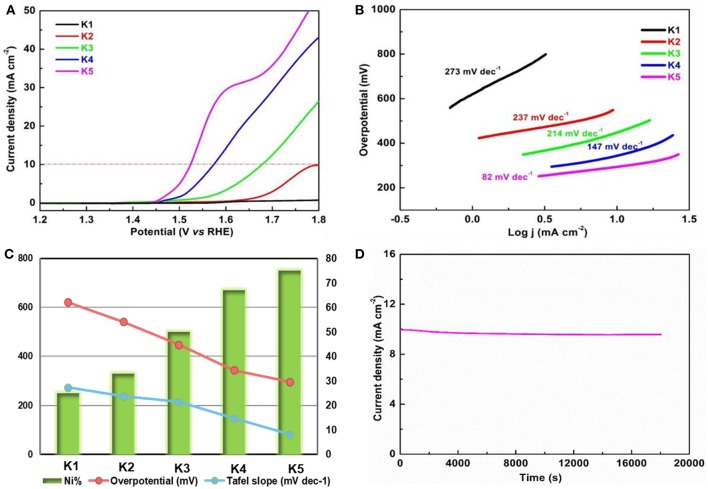
**(A)** LSV curves for the OER in an N_2_-saturated 1.0 M KOH electrolyte at 5 mV s^−1^; and **(B)** Tafel slopes of the Ni/Zn-MOF (K1-K5). **(C)** Intuitive line chart of the samples (K1-K5) for comparison of overpotential and Tafel slopes. **(D)** Chronoamperometric testing of the hierarchical urchin-like Ni/Zn MOFs for 18,000 s at a static overpotential of 296 mV in an N_2_-saturated 1.0 M KOH electrolyte.

The bimetallic MOF nanomaterials in a 1.0 M KOH electrolyte (N_2_-saturated) were measured for their electrocatalytic properties toward the OER under a standard three-electrode system. Linear sweep voltammetry (LSV) is considered an efficient method to analyze the stability of an electrocatalytic process. As shown in Figure 3A, the LSV curves of the electrodes with Ni/Zn MOF nanomaterials are obtained at 5 mV s^−1^. Notably, the hierarchical urchin-like Ni/Zn MOFs deliver a potential of ~1.45 V vs. RHE (defined as the onset potential) at 0.1 mA cm^−2^. The overpotential of the K5 sample is 296 mV at a current density of 10 mA cm^−2^, which is lower than that of the others (K1: 621 mV, K2: 541 mV, K3: 448 mV, K4: 344 mV). Moreover, the performance evaluation of the OER depends on a significant parameter (a working potential at a current density of 10 mA cm^−2^). As shown in Figure 3B, the order of the Tafel slopes is K1 > K2 > K3 > K4 > K5. The Tafel slope for the sample of the K5 catalyst is 82 mV dec^−1^, which is lower than that of the others. Obviously, these results illustrate that the overpotentials and Tafel slopes of the Ni/Zn MOFs become smaller with increasing Ni content (Figure 3C). The durability of these Ni/Zn MOFs in the test of OER also has an impact on their applications as electrocatalysts for future energy conversion and storage devices. Therefore, a potentiostatic test in a KOH electrolyte (1.0 M) was carried out. We can see from the I-t curve that 96.2% of the initial current density is retained after 18 000 s of continuous testing (Figure 3D), which can be attributed to the mass loss of the catalyst on the working electrode during the long-term potentiostatic test.

A typical parameter called electrochemical double-layer capacitance (C_dl_) is used to reasonably express the electrochemical surface area. Figure S8 shows that the K5 sample has a large C_dl_ value (12.53 mF cm^−2^), which is higher than that of the other samples (K1: 7.83 mF cm^−2^, K2: 8.84 mF cm^−2^, K3: 9.36 mF cm^−2^, and K4: 9.88 mF cm^−2^). The higher the C_dl_ value is, the greater the roughness of the electrode, and the greater the number of active sites in the nanomaterial, suggesting that the hierarchical urchin-like nanomaterials contribute greatly to the development of the catalytic reaction. Electrochemical impedance spectroscopy illustrates that the sample of K5 exhibits a much smaller charge transfer resistance than the other samples (Figure S9), revealing that a faster charge transfer occurs with increasing Ni content in Ni/Zn MOF materials. To analyze the surface areas and pore sizes of the hierarchical urchin-like Ni/Zn MOFs, the isotherms of N_2_ adsorption-desorption and Barrett-Joyner-Halenda pore size distribution tests have been conducted (Figures S10, S11). These tests present that the higher the Ni content in the Ni/Zn MOFs is, the larger the Brunauer-Emmett-Teller (BET) specific surface area, and the greater pore size distribution. The BET specific surface area of the K5 sample is 113 m^2^ g^−1^, and the pore size distribution of the K5 sample is approximately 7 nm. This nanomaterial shows better catalytic properties toward the OER because it has a greater number of active sites, which benefit from the high specific surface area and porous structure.

## Conclusions

Summarily, this study shows a simple and effective hydrothermal strategy to prepare a series of bimetallic Ni/Zn MOFs, which serve as efficient electrocatalysts for the applications of OER. These bimetallic Ni/Zn MOFs exhibited increasing electrocatalytic activity and stability with an increasing Ni content. The urchin-like microsphere structure can reduce the transport resistance of ions and facilitate the diffusion of gases to improve the utilization efficiency of the active species due to its large interfacial area and convenient diffusion channels. We hope that our work will advance the development of MOF-based electrocatalysts and pave the way for the evolution of bimetallic nanomaterials in a diverse range of energy areas such as water splitting devices, metal-air batteries, fuel cell, and other significant energy systems.

## Data Availability

The raw data supporting the conclusions of this manuscript will be made available by the authors, without undue reservation, to any qualified researcher.

## Author Contributions

YT and SZ conducted all the major experiments, designed the study and wrote the manuscript. HX and HP provided valuable inputs for the study's development and helped with manuscript writing. All authors agree to be accountable for the content of the work.

### Conflict of Interest Statement

The authors declare that the research was conducted in the absence of any commercial or financial relationships that could be construed as a potential conflict of interest.
