# Supplementary material for: Regulation of the Ni2+ Content in a Hierarchical Urchin-Like MOF for High-Performance Electrocatalytic Oxygen Evolution
Source: Front Chem. 2019 Jun 5;7:411. doi: 10.3389/fchem.2019.00411 (PMC6561350; doi:10.3389/fchem.2019.00411)

***Supplementary Material***

# 1 Supplementary Data

**Experimental Section**

*Materials and reagents*

All chemicals, Zn(NO_3_)_2_·6H_2_O, Ni(NO_3_)_2_·6H_2_O, *N,N-*dimethylformamide (DMF), ethylene glycol (CH_2_OH)_2_, p-phthalic acid (PTA, 99%), platinum carbon black, and ethanol (C_2_H_5_OH), were purchased from Shanghai Sinopharm Chemical Reagent and used without further treatment or purification. All aqueous solutions were prepared with high-purity de-ionized water (DI-water, resistance 18 MΩ cm^-1^).

*Preparation of Ni/Zn MOFs (K1-K5)*

The nanobelt-assembled hierarchical urchin-like bimetallic Ni/Zn MOFs were synthesized by a facile one-step solvothermal method. Typically, PTA (0.03 g), Ni(NO_3_)_2_·6H_2_O and Zn(NO_3_)_2_·6H_2_O with a ratio of 1 : 3 (0.05 g: 0.15 g), 1 : 2 (0.05 g: 0.10 g), 1 : 1(0.05 g : 0.05 g), 2 : 1 (0.10 g: 0.05 g) and 3 : 1 (0.15 g: 0.05 g) were dissolved in a mixed solvent of 5 mL (CH_2_OH)_2_ and 8 mL DMF with stirring for 1 h at room temperature. Then, the resulting solution was transferred into a 40 mL Teflon-lined stainless-steel autoclave. Afer sealing, the autoclave was maintained for 6 h at 150 °C. After naturally cooling to room temperature, the obtained precipitate was thoroughly washed several times with DMF and C_2_H_5_OH to remove impurities.

*Preparation of the modified electrodes and electrochemical measurements*

The obtained samples (K1-K5) were suspended in a 1% Nafion solution with concentration of 4 mg mL^-1^. Before modification, the glassy carbon electrode with diameter of 3 mm was polished with a 0.3 µm Al_2_O_3_ slurry, and then ultrasonically cleaned *via* water and ethanol. Then, 5 µL of the above suspension was added onto the glassy carbon electrode surface and dried naturally. The loading density of catalyst was confirmed to be about 0.28 mg cm^−2^. Electrochemical measurements were carried out on an electrochemical working station (CHI 760E,CH Instruments, Shanghai, China). A conventional three-electrode system was widely applied for electrochemical measurements, which consisted of a modified GCE electrode as the working electrode, an Hg/HgO electrode as the reference electrode, and carbon rod as the auxiliary electrode. All potential measurements were converted to the RHE based on the following formula Evs RHE = Evs Hg/HgO + E^θ^ Hg/HgO + 0.059 pH. Before formal electrochemical tests, the working electrodes were preconditioned by 10 cycles of cyclic voltammetric scans between 0.7-2.1 V (vs RHE). All linear sweep voltammetry (LSV) tests were performed at a scan rate of 5 mV s^-1^. Electrochemical impedance spectroscopy (EIS) measurements were performed at open circuit voltage in the frequency range of 100 kHZ to 0.01 Hz in 1 M KOH. Furthermore, the Tafel slope of these samples was obtained by ftting the experimental data with the equation η = a+blog |j|, where η is the overpotential, b is the Tafel slope, and j is the current density.

# 2 Supplementary Figures and Tables

## 2.1 Supplementary Figures


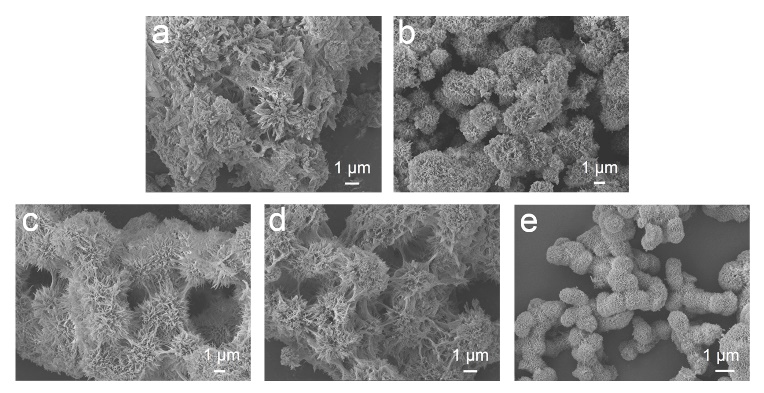


**Figure S1.** SEM (scale bar 1 μm) images of (a) K1, (b) K2, (c) K3, (d) K4, and (e) K5.


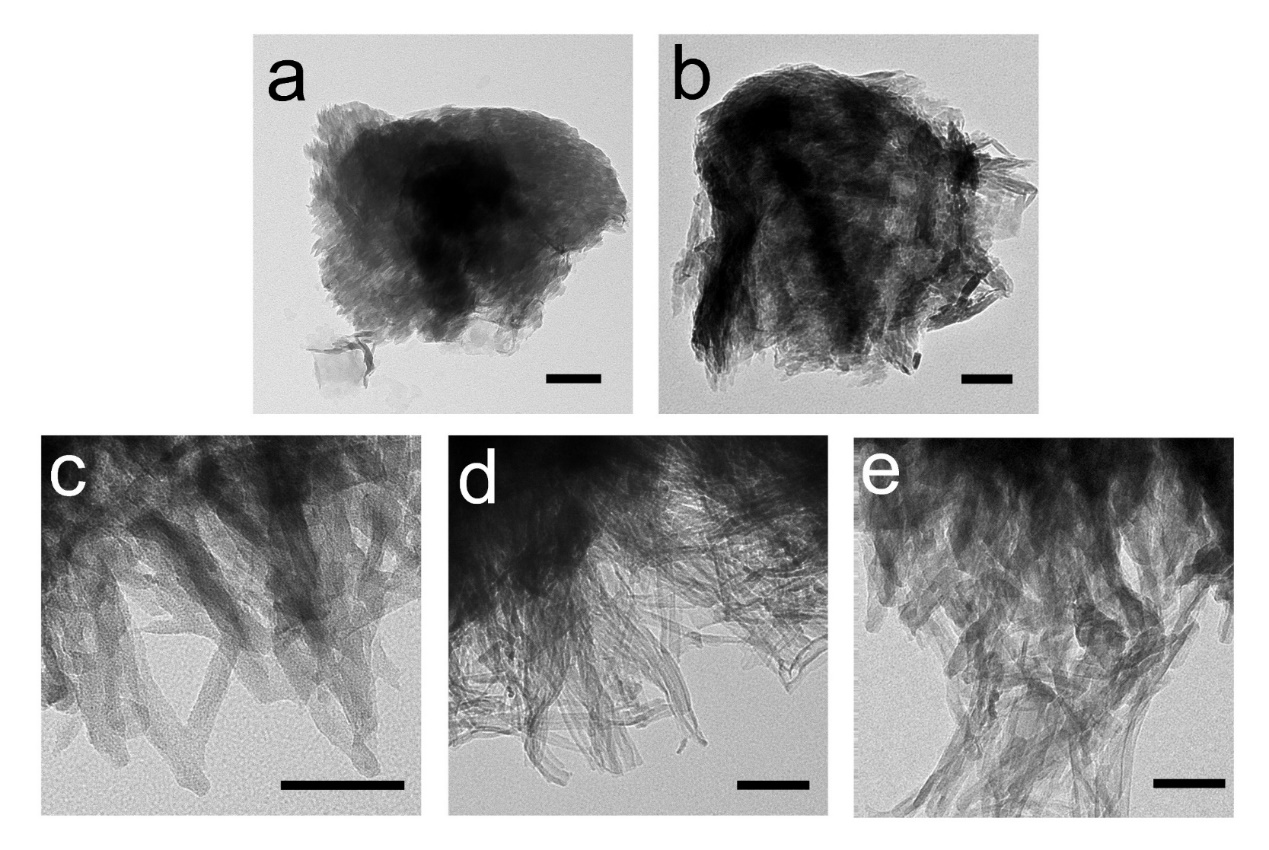


**Figure S2.** TEM (scale bar 100 nm) images of (a) K1, (b) K2, (c) K3, (d) K4, and (e) K5.


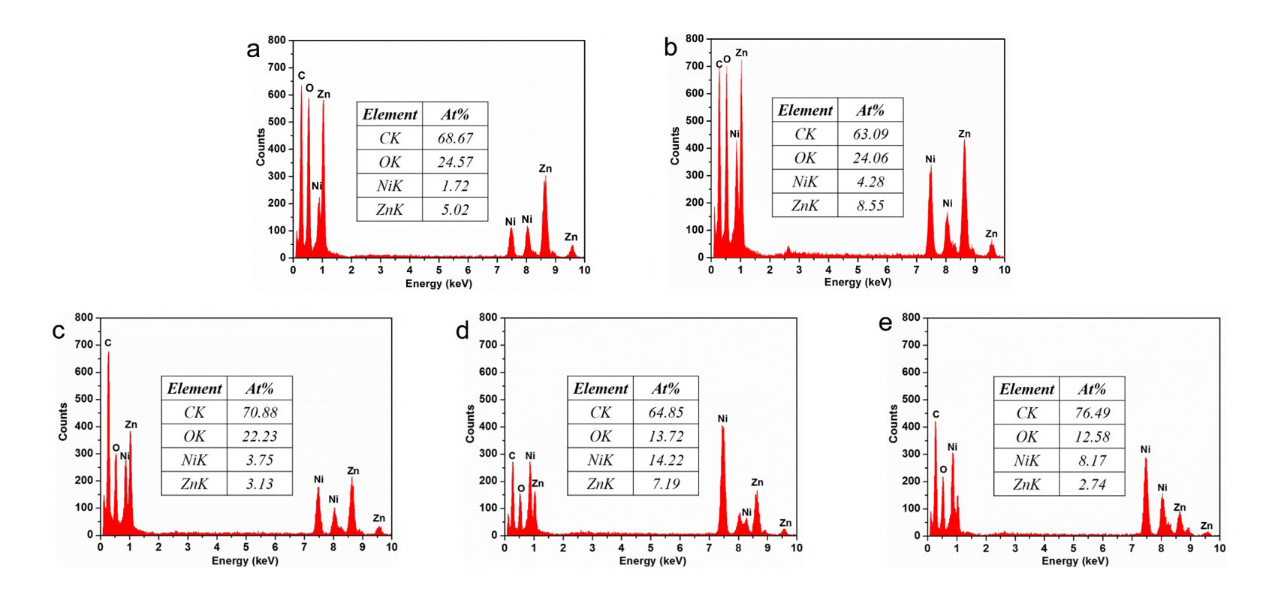


**Figure S3.** EDS analysis of: (a) K1, (b) K2, (c) K3, (d) K4, (e) K5.



**Figure S4.** XRD patterns of the K1-K5 sample.





**Figure S5.** IR spectra of the K1-K5 sample.





**Figure S6.** Ni 2p XPS spectra of K1-K5.


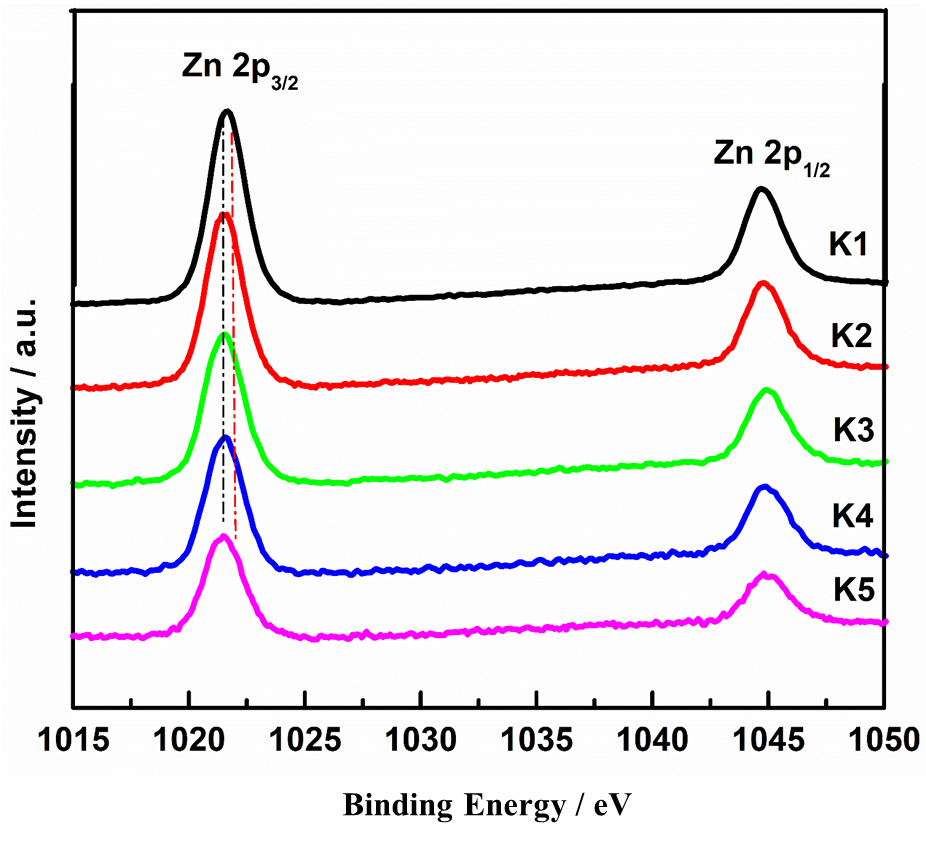


**Figure S7.** Zn 2p XPS spectra of K1-K5.


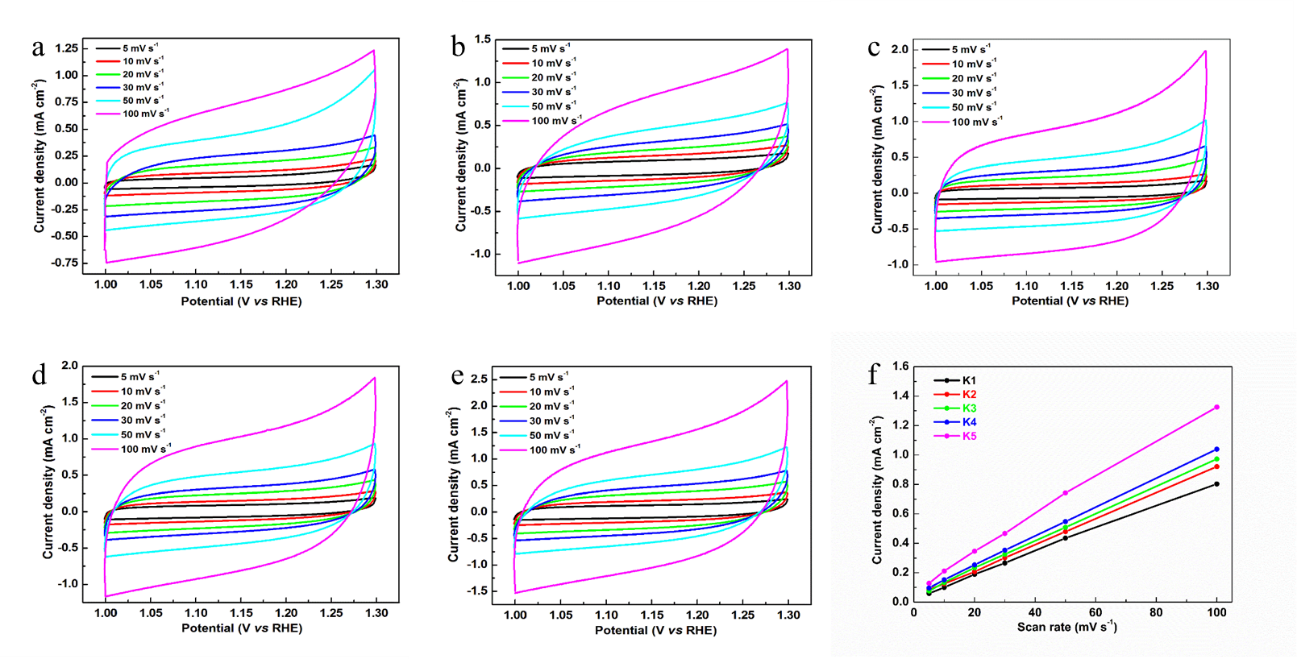


**Figure S8.** Cyclic voltammograms of (a) K1, (b) K2, (c) K3, (d) K4, and (e) K5. (f) Estimating the C_dl_ and relative electrochemically active surface areas.


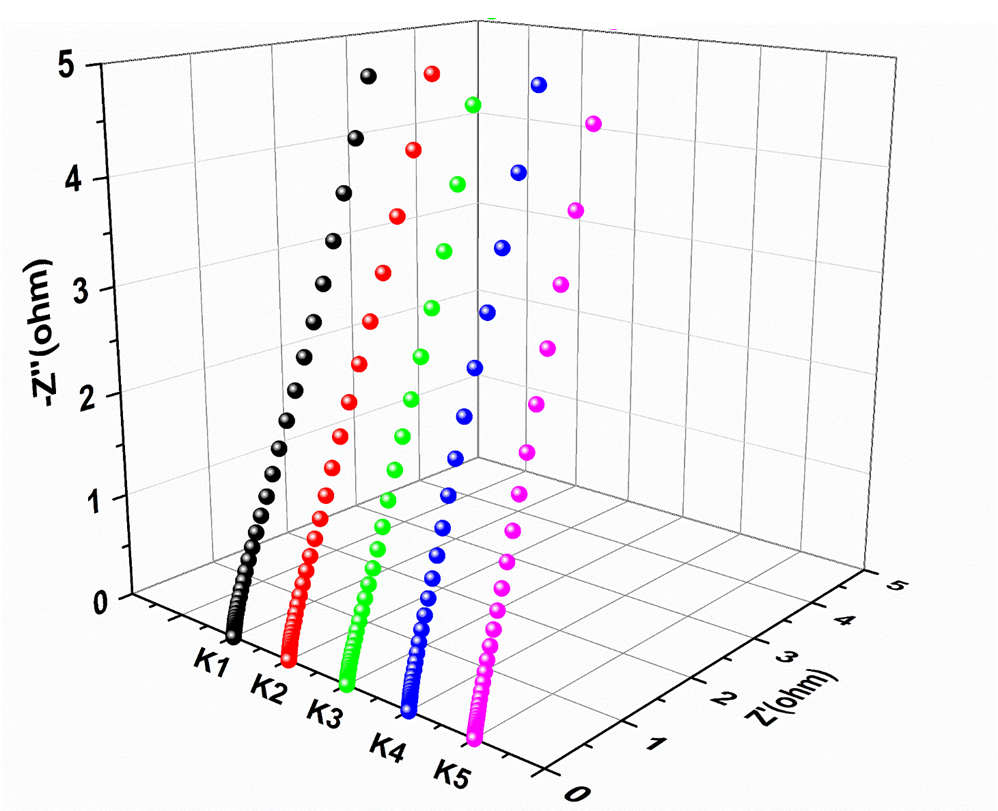
**Figure S9.** Nyquist plots of K1-K5 samples in a frequency range from 100000 to 0.1 Hz.


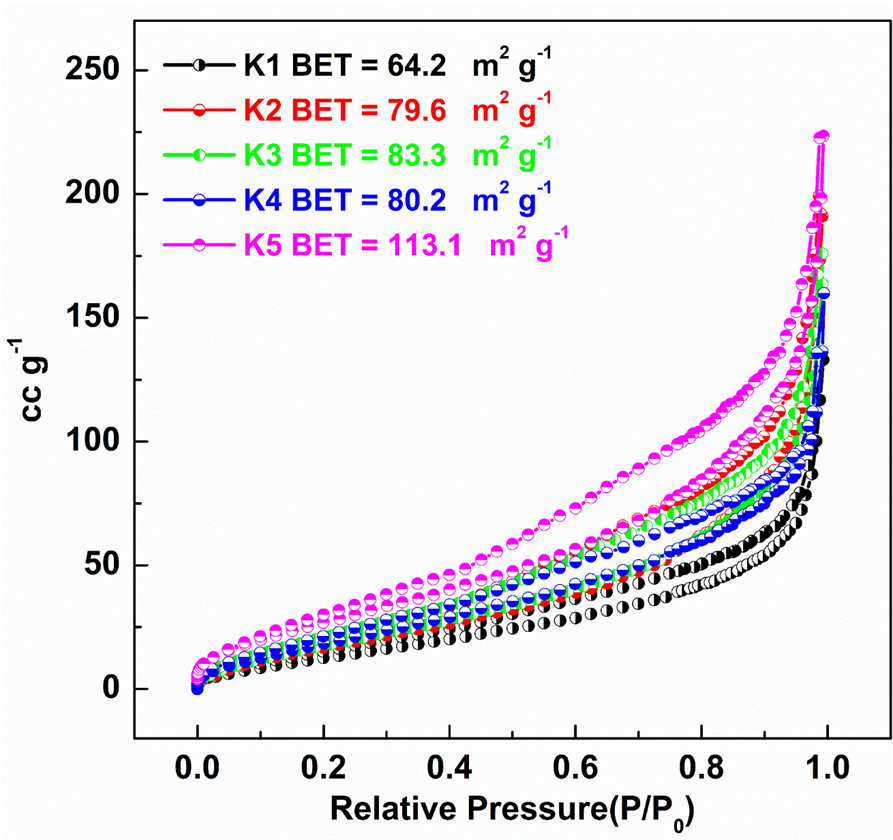
**Figure S10.** N_2_ adsorption-desorption isotherms of hierarchical urchin-like Ni/Zn MOFs.


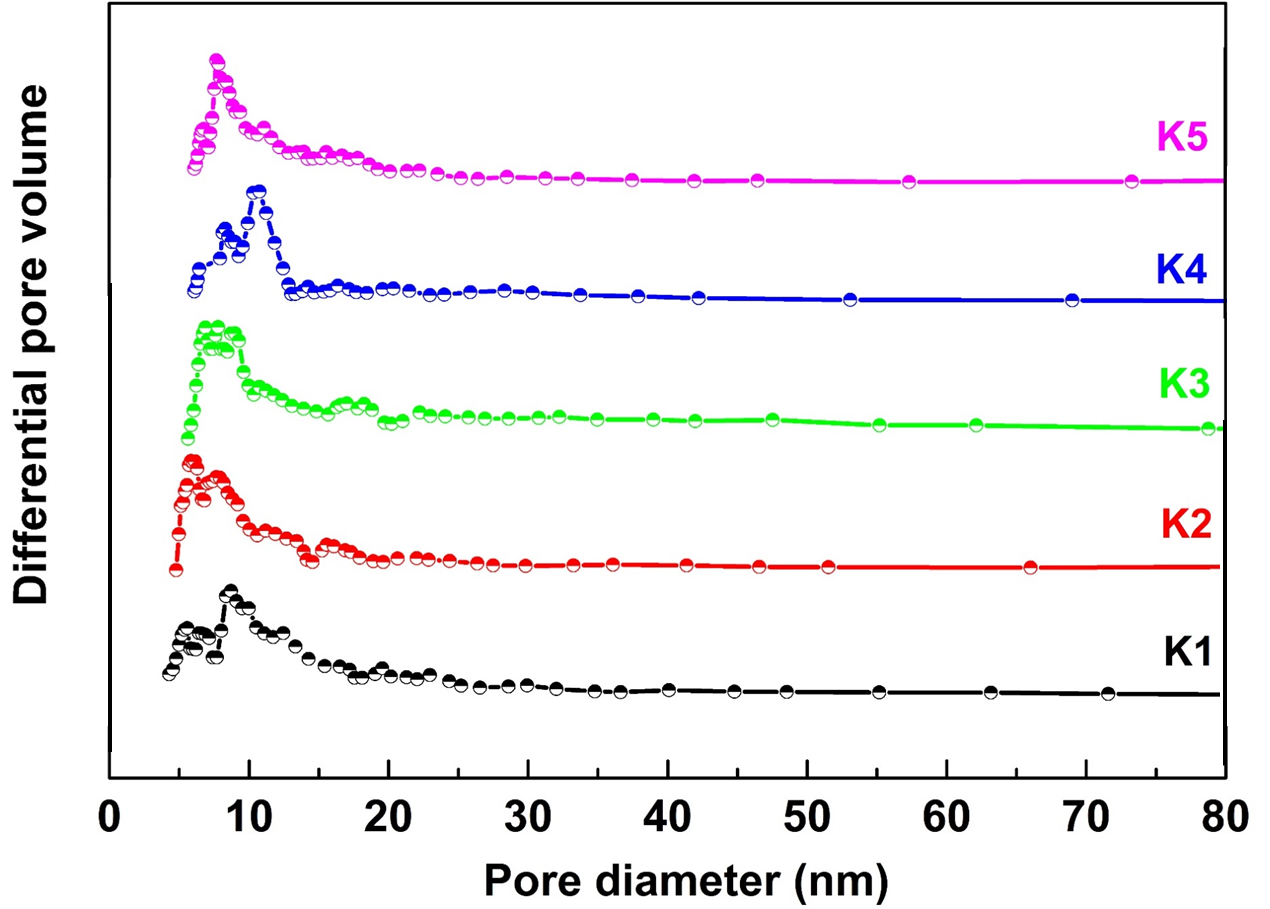


**Figure S11.** The Barrett-Joyner-Halenda pore size distribution of hierarchical urchin-like Ni/Zn MOFs.

## 2.2 Supplementary Table

**Table S1.** Ni/Zn ratios in reactants and in mixed-metal MOF samples.


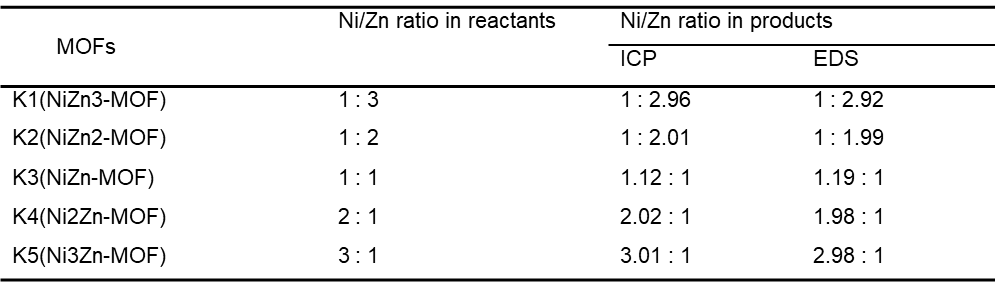

Supplement: Supplementary file 1 [file Data_Sheet_1.docx]
